# Much caution does no harm! Organophosphate poisoning often causes pancreatitis

**DOI:** 10.1186/s40560-015-0088-1

**Published:** 2015-04-30

**Authors:** Shozo Yoshida, Hideshi Okada, Shiho Nakano, Kunihiro Shirai, Toshiyuki Yuhara, Hiromasa Kojima, Tomoaki Doi, Hisaaki Kato, Kodai Suzuki, Kentaro Morishita, Eiji Murakami, Hiroaki Ushikoshi, Izumi Toyoda, Shinji Ogura

**Affiliations:** Advanced Critical Care Center, Gifu University Hospital, Gifu, Japan; ME Center, Gifu University Hospital, Gifu, Japan; Department of Emergency and Disaster Medicine, Gifu University Graduate School of Medicine, 1-1 Yanagido, Gifu, 501-1194 Japan

**Keywords:** Organophosphate poisoning (OP), Pancreatitis, Hemodiafiltration (HDF)

## Abstract

Organophosphate poisoning (OP) results in various poisoning symptoms due to its strong inhibitory effect on cholinesterase. One of the occasional complications of OP is pancreatitis.

A 62-year-old woman drank alcohol and went home at midnight. After she quarreled with her husband and drank 100 ml of malathion, a parasympathomimetic organophosphate that binds irreversibly to cholinesterase, she was transported to our hospital in an ambulance. On admission, activated charcoal, magnesium citrate, and pralidoxime methiodide (PAM) were used for decontamination after gastric lavage.

Abdominal computed tomography detected edema of the small intestine and colon with doubtful bowel ischemia, and acute pancreatitis was suspected. Arterial blood gas analysis revealed severe lactic acidosis. The Ranson score was 6 and the APACHE II (Acute Physiology and Chronic Health Evaluation) score was 14. Based on these findings, severe acute pancreatitis was diagnosed. One day after admission, hemodiafiltration (HDF) was started for the treatment of acute pancreatitis. On the third hospital day, OP symptoms were exacerbated, with muscarinic manifestations including bradycardia and hypersalivation and decreased plasma cholinesterase activity. Atropine was given and the symptoms improved. The patient’s general condition including hemodynamic status improved. Pancreatitis was attenuated by 5 days of HDF. Ultimately, it took 14 days for acute pancreatitis to improve, and the patient discharged on hospital day 32.

Generally, acute pancreatitis associated with OP is mild. In fact, one previous report showed that the influence of organophosphates on the pancreas disappears in approximately 72 hours, and complicated acute pancreatitis often improves in 4–5 days. However, it was necessary to treat pancreatitis for more than 2 weeks in this case. Therefore, organophosphate-associated pancreatitis due to malathion is more severe. Although OP sometime causes severe necrotic pancreatitis or pancreatic pseudocysts, it was thought that the present patient had a good clinical course without these complications due to the appropriate intensive care including nafamostat, antibiotics, fluid resuscitation, and HDF. In conclusion, OP-associated pancreatitis requires careful assessment because it may be aggravated, as in this case.

## Background

Organophosphates are widely used as agricultural chemicals; however, they have a high degree of human toxicity. Organophosphate poisoning (OP) is associated with multiple complications. One rare complication is acute pancreatitis. Pancreatitis due to OP is caused by increased pressure within the pancreatic duct as a result of increased exocrine secretion of pancreatic fluid [1-4]. Generally, acute pancreatitis due to OP is mild. In fact, one previous report showed that the effect of organophosphates on the pancreas disappears in approximately 72 hours, [1] and complicated acute pancreatitis often improves in 3–5 days [2,5,6]. However, herein, we report a severe case of acute pancreatitis requiring more than 2 weeks of treatment that developed as a result of OP with malathion that improved with hemodiafiltration (HDF).

## Case presentation

A 62-year-old woman drank alcohol and went home at midnight. After she quarreled with her husband and drank 100 ml of malathion, a parasympathomimetic organophosphate that binds irreversibly to cholinesterase, she was transported to our hospital in an ambulance.

On arrival her Glasgow Coma Scale score was 11 (eye, 3; verbal, 2; motor, 6). Both pupils were 2 mm, and pupillary light reflexes were absent. Physical examination revealed a body temperature of 33.8°C, tachypnea with a respiratory rate of 22 breaths/min, tachycardia with a heart rate of 102 beats/min, and normal hemodynamic parameters with a blood pressure of 128/62 mmHg. She had hyperhidrosis, cold extremities, fecal incontinence, and vomitus around the mouth. On auscultation, coarse crackles in the lung fields were detected. There were no murmurs. Bowel sounds were hyperactive.

Laboratory investigations revealed an inflammatory process with severe pancreatic impairment (Table 1). The white blood cell count was 16.2 × 10^3^/ul. The serum amylase level was high, at 596 IU/l. Serum lipase, trypsin, phospholipase A2 (PLA2), and elastase-1 were 435 IU/l, 900 IU/l, 910 ng/ml, and 1,128 ng/ml, respectively. Cholinesterase was 25 IU/l. Acute pancreatitis due to OP was diagnosed based on these results.

**Table 1:** **Laboratory findings at the time of admission**

		**<Biochemistry>**	
<CBC>		Total protein	8.2 g/dl
White blood cell	16,230/ul	Albumin	5.2 g/dl
Red blood cell	456 × 10^6^/ul	Aspartate transaminase	37 IU/l
Hemoglobin	14.6 dl	Alalime transaminase	30 IU/l
Hematocrit	42.3%	Lactate dehydrogenase	517 IU/l
Platelet	33.1 × 10^4^ ul	Alkaline phosphatase	248 IU/l
		Cholinesterase	25 IU/l
<Coagulation Status>		Creatinine	0.37 mg/dl
Activated partial thromboplastin time	20.9 sec	Blue urea nitrogen	11.4 mg/dl
Prothrombin time (PT)	>120%	Total bilirubin	0.9 mg/dl
PT-international normalized ratio	0.83	Na	137 mEq/l
Fibrinogen	328 mg/dl	K	4.3 mEq/l
		Cl	99 mEq/l
<Arterial blood gas>		C-reactive protein	0.55 mg/dl
Under the intubation		Blood glucose	220 mg/dl
FiO_2_	1	Amylase	596 IU/l
pH	7.33	Pancreas-amylase	221 IU/l
PaCO_2_	32 mmHg	Lipase	435 IU/l
PaO_2_	241 mmHg	Trypsin	>900 ng/ml
HCO_3_-	16.4 mmol/l	Phospholipase A2	910 ng/ml
Base excess	−8.1	Elasterse-1	1128 ng/ml
Lactate	65 mg/dl	Pancreatic secretory trypsin inhibitor	9.6 ng/ml

**Figure 1:**
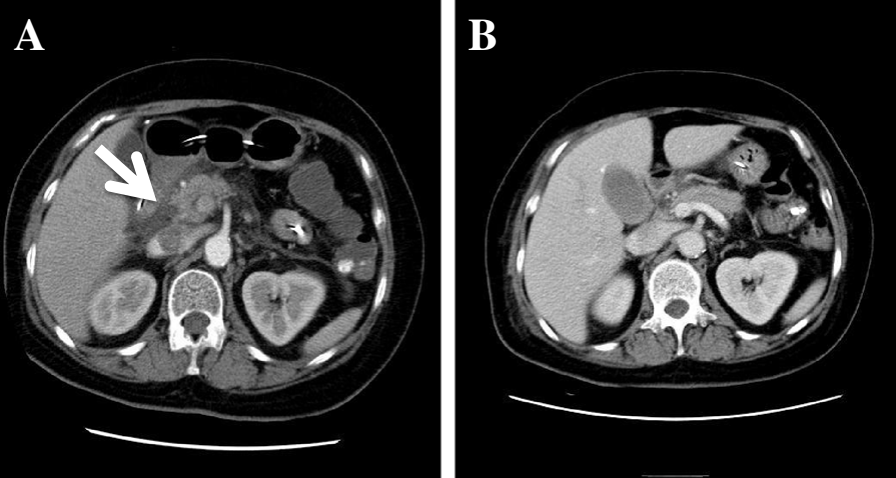
Enhanced abdominal CT scan findings. **(A)** Enhanced abdominal CT on admission. Around the head of the pancreas, the density of the adipose tissue was increased. There was a fluid collection but no swelling of the pancreas (arrow). **(B)** Abdominal CT on hospital day 11. The fluid collection has disappeared and there were no cystic changes in the pancreas.

To determine the stomach contents before gastric lavage, enhanced computed tomography (CT) of the abdomen was performed (Figure 1A). It revealed a high-density area in the stomach, which was thought to represent the bundle of the organophosphate. Likewise, wall thickening was detected from the jejunum to the ascending colon. In particular, the wall of the ascending colon was edematous, which may have been caused by OP. Moreover, the density of adipose tissue around the head of the pancreas was increased and there was a fluid collection without swelling of the pancreas. In addition, since the Ranson score [7] was 6 and the APACHE II (Acute Physiology and Chronic Health Evaluation) score [8] was 14, severe acute pancreatitis was diagnosed.

There was atelectasis of the left lower lobe of the lung and consolidation in the subpleural space of the inferior lingular segment and in the dorsal subpleural space of the right lower lobe. These findings suggested aspiration pneumonia. Non-occlusive mesenteric ischemia due to OP was also suspected based on CT findings.

As first-line therapy, gastric lavage was performed with fluid and catecholamine infusion under mechanical ventilation. In addition, activated charcoal and pralidoxime methiodide (PAM) were used for decontamination after gastric lavage. Next, since the patient experienced seizures, low-dose intravenous diazepam was administered and the seizures stopped. Arterial blood gas analysis on 100% oxygen revealed pH of 7.33, PaO_2_ of 241 mmHg, PaCO_2_ of 32.0 mmHg, HCO_3_^−^ of 16.4 mmol/l, base excess of −8.10 mmol/l, and lactate of 65 mg/dl. After sodium bicarbonate and vitamin B_1_ were administered, the acidosis improved (pH 7.4, base excess −0.2 mmol/l).

**Figure 2:**
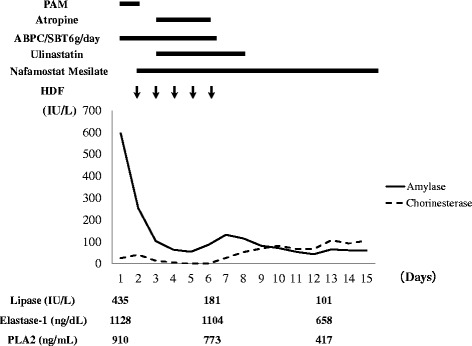
Clinical Course. PAM: pralidoxime methiodide, ABPC/SBT: Sulbactam/Ampicillin, PLA2: Phospholipase A2, HDF: Hemodiafiltration.

In addition to nafamostat mesilate and ulinastatin therapy, HDF to treat the severe acute pancreatitis was started on hospital day 2 (Figure 2). HDF was carried out using a polysulfone high-performance membrane (APS-15E, Asahi Kasei Medical, Tokyo, Japan) for 8 hours daily. Blood flow, dialysate flow, and filtrate flow rates were kept at 200 ml/min, 300–500 ml/min, and 25 ml/kg/daily, respectively. Sublood-BS (Fuso Pharmaceutical, Osaka, Japan) was used as the dialysate. After HDF treatment for 5 days, plasma lipase, elastase-1, PLA2, and CRP levels were decreased, and there was no necrosis detected in the pancreas on CT on hospital day 6.

In addition, the heart rate increased and hypersalivation was improved after the initiation of HDF. For aspiration-related pneumonia, sulbactam/ampicillin was used. After the white blood cell count and CRP decreased, HDF was stopped on hospital day 6. On hospital day 11, enhanced CT showed resolution of the fluid collection and no cystic changes in the pancreas (Figure 1B). On hospital day 14, plasma lipase, elastase-1, and PLA2 were decreased compared to those on admission. Paralytic ileus due to decreasing blood perfusion of intestinal tract, which was thought to be caused by OP, improved by hospital day 21, and oral ingestion was started. This patient was discharged on hospital day 32.

### Discussion

Malathion is a pesticide that is widely used in agriculture, residential landscaping, public recreation areas, and public health pest control programs such as mosquito eradication. Malathion itself has low toxicity; however, absorption or ingestion in humans readily results in its metabolism to malaoxon, which is substantially more toxic [9].

In studies of the effects of long-term exposure by oral ingestion of malaoxon in rats, malaoxon has been shown to be 61 times more toxic than malathion [9]. Clinical manifestations caused by organophosphates include hypersalivation, abdominal pain, nausea, vomiting, diarrhea, muscle fasciculations, bradycardia, and hypotension. In severe case, seizures, respiratory failure, shock, and death may result [10,11]. Generally, immediate treatment of OP with atropine, which blocks acetylcholine activity, is dramatically effective. Acute pancreatitis is a rare complication of OP, caused by the facilitation of exocrine secretion from the pancreas and increased internal pressure of the pancreatic duct due to the sphincter of Oddi contraction [1]. Subsequently, release of excessive acetylcholine due to the organophosphate occludes the ampulla of Vater and the pancreatic duct functionally and stimulates pancreatic acinar cells, resulting in interstitial pancreatitis [1].

Hyperamylasemia due to OP is caused not only by pancreatitis but also by intestinal ischemia, enteritis, and hypersalivation due to the direct action of the organophosphate. In fact, in previous reports, acute pancreatitis was a complication in 5.7%–29% of OP patients, [3,6] while hyperamylasemia was detected in 22%–60% of these patients [2,3,6]. Serum amylase concentration does not reflect the severity of pancreatitis although it is useful for monitoring.

Therefore, it is necessary to establish the diagnosis of pancreatitis immediately through imaging (e.g., CT), when hyperamylasemia is detected in patients with OP [12].

It was previously reported that the influence of organophosphates on the pancreas resolves within 72 hours and complicated acute pancreatitis often resolves in 3–5 days [1,2,5,6]. However, other reports suggest that OP is associated with severe necrotic pancreatitis or pancreatic pseudocysts [11,13,14]. In other words, these reports suggest that there is a spectrum of clinical findings in acute pancreatitis associated with acute OP.

Although malathion is an insecticide of relatively low human toxicity, in the present case, acute pancreatitis caused by OP was considered very severe, since the Ranson score was 6 and the APACHE II score was 14 on admission, and it took approximately 14 days for improvement of the pancreatitis despite being diagnosed by CT and treatment being initiated immediately on admission. HDF for severe acute pancreatitis keeps inflammation localized and prevents progression to multiple organ failure. Therefore, it was thought that the present patient had a good clinical course without complications such as necrotic pancreatitis and pancreatic pseudocysts due to not only nafamostat, antibiotics, and fluid resuscitation but also HDF treatment. In the present case, after HDF was started, bradycardia improved. Although the molecular weight of malathion is 330.3, relatively low, malathion is water insoluble and lipophilic. Therefore, malathion is removed very slowly through HDF. On the other hand, acute pancreatitis is frequently associated with electrocardiographic abnormalities, including arrhythmia and repolarization [15]. One previous study showed that patients with severe acute pancreatitis presented with bradycardia during hospitalization [16]. Of course, it was thought that electrolyte abnormalities and metabolic acidosis due to OP have significant influences for bradycardia in this case.

HDF treatment was not for OP but for acute pancreatitis. Presumably, bradycardia resolved with improving clinical status due to HDF. Previous report suggested that bradycardia was caused by acute pancreatitis [15,16]. One of the hypotheses could be the attack of the innervation parasympathetic of the pancreas by the castings of necrosis [15]. Also, Lambert allocated disturbances of the rhythm of pancreatic origin in two phenomena: the association of an increase in the blood rates of enzymes proteolysis, and a hyperactivity of vagus nerve [16]. HDF decreased these factors and subsequently improved clinical status.

Therefore, bradycardia in this case may have been caused not only by electrolyte abnormalities and metabolic acidosis due to organophosphate poisoning but also by acute pancreatitis instead of OP, and HDF may be effective for bradycardia.

## Conclusion

OP is associated with various complications, including acute pancreatitis. Therefore, it is necessary to carefully examine and assess patients with acute OP.

## Consent

The patient gave written informed consent for publication of this case report and all accompanying images. A copy of the consent form is available for review by the Editor-in-Chief.
